# The brief overview, antivirus and anti-SARS-CoV-2 activity, quantitative methods, and pharmacokinetics of cepharanthine: a potential small-molecule drug against COVID-19

**DOI:** 10.3389/fphar.2023.1098972

**Published:** 2023-07-31

**Authors:** Binbin Xia, Li Zheng, Yali Li, Wenfang Sun, Yang Liu, Liushui Li, Jingyao Pang, Jing Chen, Jiaxin Li, Hua Cheng

**Affiliations:** ^1^ Department of Pharmacy, Beijing Luhe Hospital Affiliated to Capital Medical University, Beijing, China; ^2^ Department of Pharmacy, China Aerospace Science & Industry Corporation 731 Hospital, Beijing, China; ^3^ Department of Pharmacy, Beijing Hospital, National Center of Gerontology, Beijing, China

**Keywords:** cepharanthine, antivirus, anti-SARS-CoV-2, pharmacokinetics, research progress

## Abstract

To effectively respond to severe acute respiratory syndrome coronavirus 2 (SARS-CoV-2), an increasing number of researchers are focusing on the antiviral activity of cepharanthine (CEP), which is a clinically approved drug being used for over 70 years. This review aims to provide a brief overview of CEP and summarize its recent findings in quantitative analysis, pharmacokinetics, therapeutic potential, and mechanism in antiviral and anti-SARS-CoV-2 activity. Given its remarkable capacity against SARS-CoV-2 infection *in vitro* and *in vivo*, with its primary target organ being the lungs, and its good pharmacokinetic profile; mature and stable manufacturing technique; and its advantages of safety, effectiveness, and accessibility, CEP has become a promising drug candidate for treating COVID-19 despite being an old drug.

## 1 Introduction

The emergence of the corona virus disease 2019 (COVID-19) triggered an unprecedented challenge to public health and human life worldwide since December 2019. This illness is caused by a novel coronavirus, severe acute respiratory syndrome coronavirus 2 (SARS-CoV-2), and presents as a severe acute respiratory syndrome. According to the latest statistics from the World Health Organization (WHO), over 638.17 million confirmed cases of COVID-19 and more than 6.61 million death cases have been reported globally. The COVID-19 pandemic has become the world’s largest and deadliest infectious disease outbreak, posing a serious global public health challenge that needs to be urgently addressed.

In addition to implementing a strict dynamic zero-COVID-19 policy, China prevented and treated SARS-CoV-2 mainly through vaccines (Cao et al., 2023; Fu et al., 2023), neutralizing antibodies (Leung et al., 2023; Liu et al., 2023), traditional Chinese medicines (Zhang et al., 2023; Zhao et al., 2023), natural products (Yang and Wang, 2021; Wang et al., 2022a), and small-molecule drugs (Zhang et al., 2021; Wang et al., 2022b). According to the National Health Commission of China, by 29 November 2022, more than 3.443 billion doses of the SARS-CoV-2 vaccine had been administered in China. However, the vaccine and neutralizing antibodies only target the surface structural proteins of the virus, and their efficiency can be easily affected by virus mutations. Several neutralizing antibodies granted by Emergency Use Authority (EUA) in the United States have been suspended due to their ineffectiveness against the Omicron variants of SARS-CoV-2.

The angiotensin-converting enzyme 2 (ACE2) receptor of SARS-CoV-2, which is also the surface receptor of SARS-CoV-2 spike glycoprotein, is expressed in alveolar epithelial cells and promotes the virus to enter and infect host cells (Hoffmann et al., 2020). The serine protease TMPRSS2 is used by SARS-CoV-2 for S-protein priming. However, the surface structural proteins of SARS-CoV-2 easily mutate (Meng et al., 2022), while the intracellular processes are relatively conserved and less susceptible to mutation. For instance, the Omicron spike protein of SARS-CoV-2 inefficiently uses the cellular protease TMPRSS2, promoting cell entry through the endocytic pathway (Meng et al., 2022). Thus, small-molecule medicines that act on intracellular processes may have the potential to be universally efficient against SARS-CoV-2 variants (Meng et al., 2022). Global efforts are underway to identify safe and efficient treatments for COVID-19. Unfortunately, despite these efforts, only three small-molecule drugs currently have the EUA from the United States Food and Drug Administration (FDA) for the treatment of nonhospitalized adult patients with mild-to-moderate symptoms of COVID-19:

Paxlovid^TM^ (nirmatrelvir tablets/ritonavir tablets, co-packaged), one of the 3CL protease inhibitors from Pfizer; Lagevrio^TM^ (molnupiravir capsule), one of the RNA polymerase inhibitors from Merck; and Veklury^TM^ (remdesivir injection), one of the RNA polymerase inhibitors from Gilead, are some of the drugs used in treating COVID-19. The State Food and Drug Administration (SFDA) in China conditionally approved the registration application of Azvudine tablets, the original anti-AIDS medicine, to add the indication for the treatment of COVID-19 on 25 July 2022. This became the first innovative small-molecule oral drug for the treatment of COVID-19 in China. However, information about the safety and effectiveness of these four innovative drugs is still limited in clinical use.

The high transmission and exponential growth rate of SARS-CoV-2 variants, coupled with the slow process of developing innovative drugs, have highlighted the need to quickly reuse existing drugs. This has led to the strategy of “new use of old drugs,” such as cepharanthine (CEP), as potential treatments for COVID-19. CEP is a conventional drug that has been used in Japan since 1951 to treat many acute and chronic diseases.

It was recently identified as the most effective drug against SARS-CoV-2-related pangolin coronavirus, a less pathogenic model for SARS-CoV-2, in a large drug screen of 2,406 clinically approved drugs (Fan et al., 2020). Tong et al. from Beijing University of Chemical Technology obtained the national invention patent of China for pangolin coronavirus xCoV and its application, as well as the application of drugs against the coronavirus infection (ZL 2021 1 0172158.7) in May 2022. The patent specification revealed that a low concentration of CEP (10 μmol/L) could significantly inhibit the capability of coronavirus infection at the cellular level, and the virus content decreased by 15,393 times in comparison with the control group.

Given this finding and the close homology between the genome sequences of SARS-CoV-2 and SARS-CoV-2-related pangolin coronavirus, CEP has been shown with significant potential for treating COVID-19. In order to expedite the research and development of anti-COVID-19 medication, this review aims to summarize and analyze the current findings in quantitative analysis and pharmacokinetics, as well as to discuss its therapeutic potential and mechanism in anti-COVID-19 treatment.

## 2 A brief overview and current uses of CEP

**FIGURE 1:**
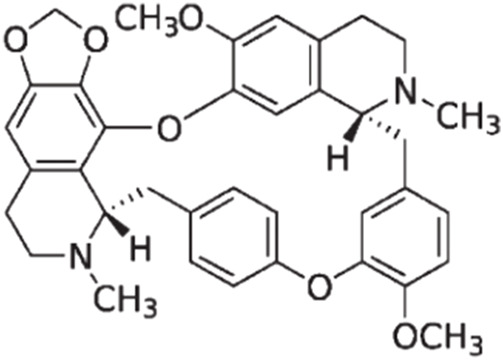
Chemical structure of cepharanthine (CEP).

CEP is a naturally occurring bisbenzylisoquinoline (BBIQ) alkaloid primarily derived from plants of genus *Stephania (Menispermaceae)* such as *Stephania japonica Miers*, *Stephania delavayi Diels*, *Stephania cepharantha Hayata*, *Epigeal Stephania Root,* and long *Stephania* herb*.* It was initially separated and purified in 1934 by a Japanese pharmacist and named after one of its key sources: *Stephania cepharantha Hayata.* Chemically, CEP belongs to the family of BBIQ cyclic alkaloids, which includes tetrandrine, dauricine, curine, trilobine, neferine, daphnoline, and berbamine. Its chemical name is 6′,12′-dimethoxy-2,2′-dimethyl-6,7-[methylenebis-(oxy)] oxyacanthan, and its molecular formula is C_37_H_38_N_2_O_6_. The molecular weight of CEP is 606.71, and its structural formula is shown in Figure 1.

CEP has been used as a conventional drug in Japan since 1951 to treat many acute and chronic diseases, including leukopenia (Nomoto et al., 2004; Bailly, 2019; Rogosnitzky et al., 2020), alopecia (Bailly, 2019; Rogosnitzky et al., 2020), malaria (Bailly, 2019), exudative middle-ear catarrh (Rogosnitzky and Danks, 2011; Rogosnitzky et al., 2020), idiopathic thrombocytopenic purpura (Nakayama et al., 1992; Rogosnitzky et al., 2020), and snake bites (Rogosnitzky and Danks, 2011; Bailly, 2019; Rogosnitzky et al., 2020). It can be administered over a long period in a relatively large dose, either orally or intravenously (Bailly, 2019). Importantly, this natural product is safe and well-tolerated, with very limited undesirable effects (Bailly, 2019). CEP was an inexpensive and well-established medication in Japan, authorized for more than 70 years. As early as 30 years ago, CEP was applied in the empirical treatment of pneumoconiosis in China, with good results (Shen et al., 2023). Presently, no fewer than four pharmaceutical enterprises in China have been approved to manufacture and distribute CEP for the prevention and treatment of cancer patients with leukopenia endangered by radiotherapy and chemotherapy.

**FIGURE 2:**
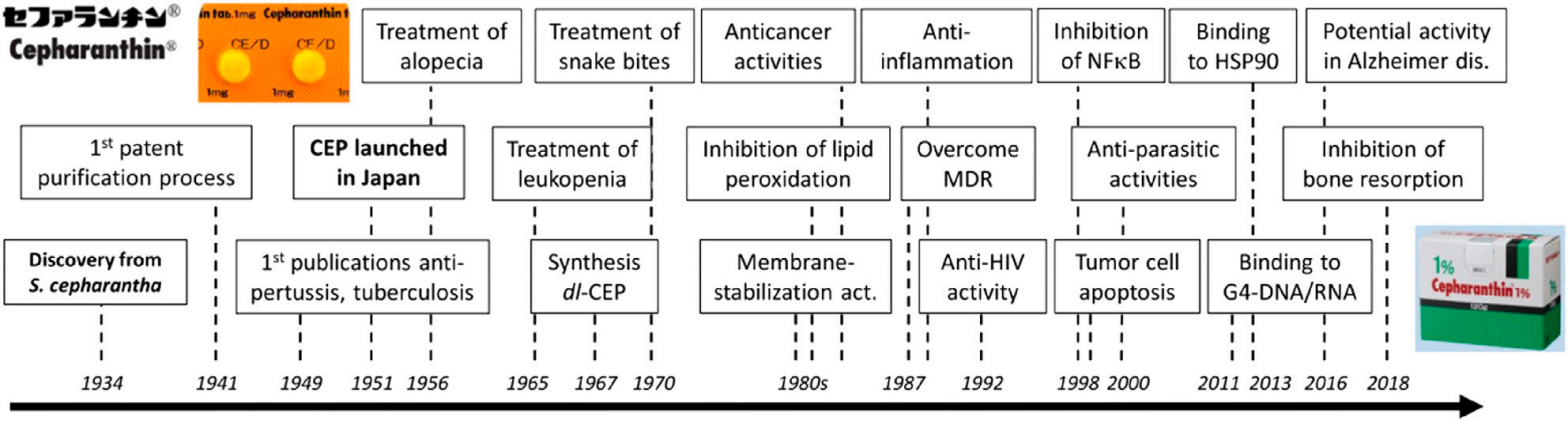
History of CEP discovery and development (Bailly, 2019).

CEP has been shown to have multiple molecular mechanisms, including stabilizing cell membrane fluidity (Matsuda et al., 2014), inhibiting drug efflux (Peng et al., 2012; Bailly, 2019), scavenging free radicals (Rogosnitzky and Danks, 2011), alleviating inflammatory factor production, inhibiting cytoplasmic nuclear transcription factor (NF-κB) (Lin et al., 2018) and activating the adenosine-activated protein kinase (AMPK) signaling pathway (Fan et al., 2020), inhibiting the integrins/ILK/RACK1/PKCα/NF-κB signaling axis (Yang et al., 2021), and inhibiting receptor activator of nuclear factor-κB (NF-κB) ligand (RANKL)-induced osteoclast formation and bone-resorbing activities (Lin et al., 2018). These mechanisms have been linked to various biological activities of CEP, such as anti-inflammatory (Bailly, 2019; Rogosnitzky et al., 2020), immunomodulatory (Yamazaki et al., 2017; Bailly, 2019), anti-osteoporotic (Zhou et al., 2018; Yao et al., 2022), antioxidant (Bailly, 2019), inhibition of drug efflux transporters (Peng et al., 2012; Kathawala et al., 2014), exerting protective effects against pulmonary fibrosis (Li et al., 2022), anticancer (Tang et al., 2018; Bailly, 2019; Rogosnitzky et al., 2020; Wang et al., 2020), and anti-parasitic effects (Bailly, 2019). A historical overview of the research and development of CEP, which traces its main clinical applications and biochemical characteristics from 1934 to 2018, is presented in Figure 2 (Bailly, 2019).

In recent years, it has been reported that CEP shows significant bioactivity against a range of viruses, including HIV-1 (Baba, 1997; Okamoto et al., 1998; Baba et al., 2001; Okamoto et al., 2001), hepatitis B virus (HBV) (Rogosnitzky et al., 2020), herpes simplex virus type 1 (HSV-1) (Liu et al., 2004; Liu et al., 2021a; Liu et al., 2021b), human T-cell lymphotropic virus (HTLV) (Toyama et al., 2012), Zika virus (ZikaV) (Zhang et al., 2022), Ebola virus (EBOV) (Zhang et al., 2022), porcine reproductive and respiratory syndrome virus (PRRSV) (Yang et al., 2021), porcine circovirus type 2 (PCV2) (Xu et al., 2020), SARS virus (SARS-CoV) (Zhang et al., 2005; Rogosnitzky et al., 2020; Wang and Yang, 2020), HCOV-OC43 (Kim et al., 2019), and other coronaviruses. The main mechanism underlying the antiviral activity of CEP is its ability to inhibit the production of inflammatory cytokines and chemokines within cells (Bailly, 2019). Accumulating evidence suggests that the AMPK-NF-κβ axis is essential to the mode of action of CEP. The activation of AMPK promotes anti-inflammatory responses in cells exposed to stress/danger signals, primarily through the inhibition of NF-κB signaling. This, in turn, boosts anti-inflammatory responses in cells exposed to stress/danger signals (Bailly, 2019). Table 1 summarizes previous investigations examining the antiviral effects of CEP and its possible mechanism, indicating its potential as a drug for managing COVID-19.

**TABLE 1:** Summary of previous investigations examining the antiviral effects of CEP and its mechanism of action.

Virus	EC_50_/IC_50_ (µg/mL)	TC_50_/CC_50_ (µg/mL)	Pharmacologic action	Mechanism of action	References
HIV-1	0.016	2.2	Dampening the virus’s pathogenicity;Crossing the blood–brain barrier and inducing neural cell death	Suppresses HIV-1 long-terminal repeat-driven gene expression by inhibiting the activation of NF-κB	Baba (1997), Okamoto et al. (1998), Baba et al. (2001), Okamoto et al. (2001)
	—	—		Suppresses the production of inflammatory cytokines and a chemokine, i.e., TNF-alpha, interleukin (IL)-1 beta, IL-6, and IL-8	Okamoto et al. (2001)
	—	—		Inhibition of the HIV-1 entry process by reducing plasma membrane fluidity	Matsuda et al. (2014)
HBV	—	—	Inhibits viral replication and suppresses viral HBeAg antigen production	Suppresses HBV via downregulation of host Hsc70 expression, thereby inhibiting viral replication and HBeAg production	Rogosnitzky et al. (2020)
HSV-1	1.56	7.52	—	—	Liu et al. (2004)
	0.835	5.4 for Vero cell; 9.0 for HeLa cell	Reducing HSV-1 infection and subsequent reproduction	Arrests the cell cycle in the G2/M phase and induces apoptosis in infected cells by inhibiting the PI3K/Akt and p38 MAPK signaling pathways	Liu et al. (2021b)
				To promote interferon-independent autophagy through the STING/TBK1/P62-mediated signaling pathways	Liu et al. (2021a)
HTLV-1	—	—	Triggers apoptosis of HTLV-1-infected cells through the caspase-dependent pathway	Inhibits NF-kB signaling pathway and suppresses viral replication and then reduces viral titer	Toyama et al. (2012)
PRRSV	—	—	Alleviation of PRRSV infection	Inhibits the expression of integrins β1 and β3, integrin-linked kinase (ILK), RACK1, and PKCα, leading to NF-κB suppression	Yang et al. (2021)
				Inhibition of integrins/ILK/RACK1/PKCα/NF-κB, and therefore downregulation of inflammatory responses	
PCV2	—	8.048 ± 0.614	CEP has a significant antiviral effect	Inhibits mitochondrial apoptosis induced by PCV2	Xu et al. (2020)
SARS -CoV	6.0–9.5	—	Inhibits viral replication	Inhibits viral RNA replication, blocks the expression of viral proteins, and suppresses production of pro-inflammatory molecules toward preventing an exacerbated cytokine response to the viral infection	Zhang et al. (2005), Rogosnitzky et al. (2020), Wang and Yang (2020)
HCoV -OC43	0.443	6.395	Inhibits viral replication and infectivity	Blocks the expression of the viral spike protein and nucleoprotein	Kim et al. (2019)
			Dampens virus-induced host response	Inhibits the binding of the spike protein to membrane receptors (9-O-acetylated sialic acid glycan-based receptors)	
				Expression of the new spike protein and nucleoproteins	

HIV-1: human immunodeficiency virus type 1; HBV: hepatitis B virus; HSV-1: herpes simplex virus type 1; HLTV-1: human T-lymphocytic virus type 1; PRRSV: porcine reproductive and respiratory syndrome virus; PCV2: porcine circovirus type 2; SARS-CoV: severe acute respiratory syndrome coronavirus; HCoV-OC43: human coronavirus type OC43.

## 3 The anti-SARS-CoV-2 activity and its potential mechanism of CEP

**FIGURE 3:**
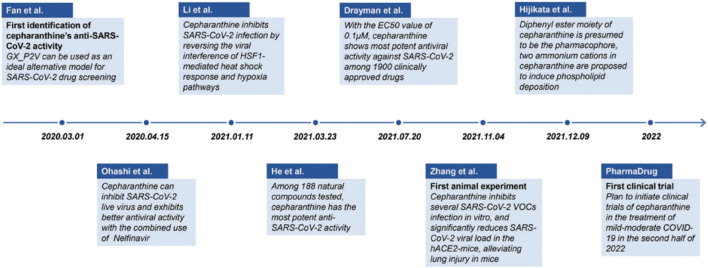
Timeline of the main events in research progress on CEP inhibition of SARS-CoV-2 (Fan et al., 2022).

CEP has recently been identified as the most efficient drug against SARS-CoV-2-related pangolin coronavirus, a less pathogenic model for SARS-CoV-2, in a large drug screening of 2,406 clinically approved drugs. Tong et al. from Beijing University of Chemical Technology obtained the national invention patent of China for pangolin coronavirus xCoV and its application, as well as the application of drugs against the coronavirus infection in May 2022. The patent specification demonstrated that a low concentration of CEP (10 μmol/L) could significantly inhibit coronavirus infection at the cellular level, with the virus content decreasing by 15,393 times compared to the control group. These findings were first published online in the National Medical Journal of China (Fan et al., 2020). A brief summary of examining the anti-SARS-CoV-2 capacity of CEP and its mechanism is shown in Table 2-A. Since then, numerous studies on the inhibitory activities of CEP against SARS-CoV-2 infection have been carried out in an orderly fashion (Figure 3).

**TABLE 2:** Summary of recent studies examining the anti-SARS-CoV-2 capacity of CEP and its potential mechanism.

NO.	Virus	Cell types	EC_50_/IC_50_ (µmol/L)	CC_50_ (µmol/L)	Mechanism of action	References	Remark
A	GX_P2V	Vero E6	0.98	39.30	Reverses most dysregulated genes and pathways in infected cells including endoplasmic reticulum stress/unfolded protein response and HSF1-mediated heat shock response	Fan et al. (2020), Li et al. (2021)	—
					Regulates the glycosylation of viral spike proteins and reduces the N-glycosylation of proteins located on the cell membrane surface	An et al. (2022)	
					May protect lymphocytes by inhibiting the infection of SARS-CoV-2 and modulating cellular stress responses and autophagy	Li et al. (2021)	
B	SARS-CoV-2	Vero E6/TMPRSS2	0.35	25.10	Inhibits SARS-CoV-2 entry through the blocking of viral binding to target cells	Ohashi et al. (2021)	
C	SARS -CoV	—	—	—	As a potential inhibitor of the SARS-CoV-2, the Nsp12–Nsp8–Nsp7 complex combines well with a multi-subunit complex of nonstructural proteins (NSP), the NSP12–NSP7–NSP8 complex, which is deemed critical for viral replication and transcription	Yin et al. (2020), Ruan et al. (2021)	
	SARS -CoV-2						
D	SARS-CoV2	BL21 (DE3)	0.4	—	Inhibits the ATPase activity of the purified recombinant SARS-CoV-2 Nsp13 protein	White et al. (2020)	Test the ATPase activity of purified recombinant
							SARS-CoV-2 Nsp13 protein from BL21 (DE3) cells
E	SARS-CoV-2	293TACE2	0.351	—	Blocks host calcium channels, thus inhibiting Ca^2+^-mediated fusion and suppressing virus entry	He et al. (2021)	S-G614 pseudovirus
		Calu-3	0.759	—			
		A549-ACE2	0.911	—			
		293T-ACE2	0.0537	—			S- D614 pseudovirus
			0.047	—			N501Y.V1 (B.1.1.7)
			0.296	—			N501Y.V2 (B.1.351)
F	SARS -CoV-2	A549 cells	0.13	—	Do not inhibit 3CL protein activity in 293T cells. The inhibitory activity of CEP against SARS-CoV-2 was not achieved by the 3CL protein	Drayman et al. (2021)	
	HCoV -OC43		0.77	—			
G	SARS-CoV-2	—	—	—	CEP showed the best binding affinity with nsp10, nsp14, nsp16, S protein, and ABL1 and exhibited good binding affinities with the 3CL protein	Hossain et al. (2022)	
H	SARS-CoV-2	Vero E6 /TMPRSS2	1.90	—	Inhibition of the Niemann–Pick type C disease-causing gene (NPC1): CEP causes disruption of cellular/lysosomal lipid homeostasis and potentially inhibits the entry, replication, and exit of SARS-CoV-2	Lyu et al. (2017), Sturley et al. (2020), Hijikata et al. (2022)	IC_90_ was 4.46µmol/L
I	SARS -CoV-2	A549	1.67	30.92	SARS-CoV-2 binds and hijacks the host factor IGF2BP1 to stabilize vRNA and enhance viral translation; knockdown of heat shock proteins (HSP90AB1, HSPA9, and HSPD1) reduced the SARS-CoV-2 vRNA levels in Huh-7.5.1 cells;	Zhang et al. (2022)	
	SARS -CoV-2 variant (B.1.351)	A549	0.24	—	CEP is a heat shock protein inhibitor		
		Huh-7.5.1	0.06	—			
	ZIKV	Huh-7	2.19	24	—		
	EBOV	Huh-7.5.1-VP30	0.42	5.08	—		

EC_50_: concentration for 50% of maximal effect; IC_50_: 50% inhibitory concentration; half-maximal [50%] inhibitory concentration; CC_50_: cytotoxicity concentration 50%; IC_90_: 90% inhibitory concentration.

**FIGURE 4:**
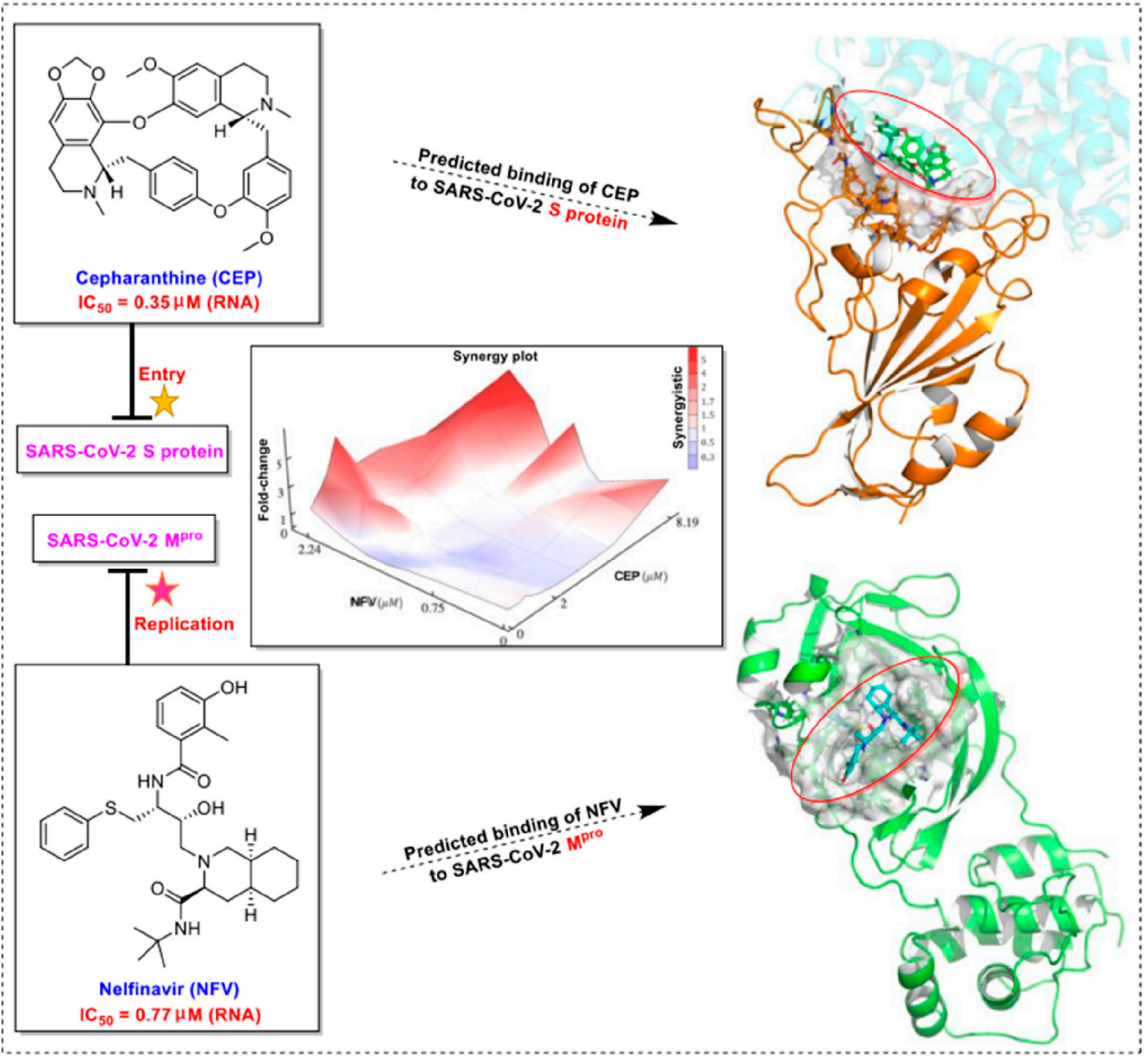
Mechanism of CEP in combination with FDA-approved nelfinavir inhibits SARS-CoV-2 (Ohashi et al., 2021).

Subsequently, Takaji Wakita et al. from the National Institute of Infectious Diseases in Japan published a similar study confirming the potential therapeutic effect of CEP on SARS-CoV-2 (Ohashi et al., 2021). In this study, they demonstrated that CEP and nelfinavir (NFV), one of the human immunodeficiency virus protease inhibitors, inhibit SARS-CoV-2 proliferation in Calu-3 cells , a human-derived lung epithelial cell line. This study also found that CEP and NFV have higher antiviral potential than remdesivir and chloroquine, which received EUA from the FDA, in Vero E6/TMPRSS2 cells (Ohashi et al., 2021). CEP inhibited SARS-CoV-2 entry by blocking viral binding to target cells, while NFV inhibited the catalytic activity of main viral protease to suppress viral replication. Both CEP and NFV, when used alone, could reduce the level of viral RNA in different modes of action, and the synergistic effect of the combined treatment in inhibiting SARS-CoV-2 proliferation was highlighted (Ohashi et al., 2021). The mechanism of CEP in combination with FDA-approved nelfinavir inhibits SARS-CoV-2, as shown in Figure 4. Additionally, the EC_50_ and CC_50_ ratio was over 70 (IC_50_ = 0.35 μmol/L, IC_90_ = 0.91 μmol/L, and CC_50_ = 25.10 μmol/L) (Table 2-B), indicating that SARS-CoV-2 inhibition could be achieved within minimal toxicity (Ohashi et al., 2021).

The NSP12–NSP7–NSP8 complex of SARS‐CoV or SARS‐CoV‐2, which was essential for viral replication and transcription, was generally regarded as a potential target for antiviral therapy (Ruan et al., 2021). In June 2020, Tai Yang et al. from the Chengdu Medical College established the NSP12–NSP7 interface model and NSP12–NSP8 interface model according to the NSP12–NSP7–NSP8 complex (PDB ID: 6NUR) structure of SARS-CoV and the NSP12–NSP7-NSP8 complex (PDB ID: 7BW4) structure of SARS-CoV-2 for binding free energy calculations based on virtual screening and docking scores from eight approved drugs (Ruan et al., 2021). The results indicated that CEP could bind well with NSP12–NSP7–NSP8 in the crystal structure, which suggests that CEP is a potential inhibitor of the SARS-CoV-2 Nsp12–Nsp8–Nsp7 complex (Table 2-C) (Ruan et al., 2021).

Mark Andrew White et al. from the University of Texas Medical Branch found that CEP exhibited the best binding activity to the ATP-binding site of SARS-CoV-2 helicase (Nsp13), which is critical for viral replication and the most conserved non-structural protein within the coronavirus family, using homology modeling and molecular dynamics approaches to screen ∼970,000 chemical compounds (White et al., 2020). *In vitro* enzyme activity experiments were conducted to test the inhibitory ability of CEP on the ATPase activity of purified recombinant SARS-CoV-2 Nsp13 protein. The IC_50_ value was 0.4 μmol/L, indicating its noteworthy inhibitory ability on ATPase activity (White et al., 2020). The significant inhibitory effect of CEP on the Nsp13 helicase may be one of the mechanisms related to its anti-SARS-CoV-2 activity (Table 2-D).

In January 2021, Tong et al. from Beijing University of Chemical Technology conducted RNA sequencing to reveal the response and antiviral activity of cells to viruses. The results further confirmed that CEP could reverse the majority of the dysfunctional genes and pathways in virus-infected cells by interfering with heat shock factor 1 (HSF1)-mediated heat shock response, endoplasmic reticulum (ER) stress/unfolded protein response, and hypoxia pathway manipulated with by the virus. This also provided evidence for CEP as a promising therapeutic drug (Table 2-A) (Li et al., 2021).

**FIGURE 5:**
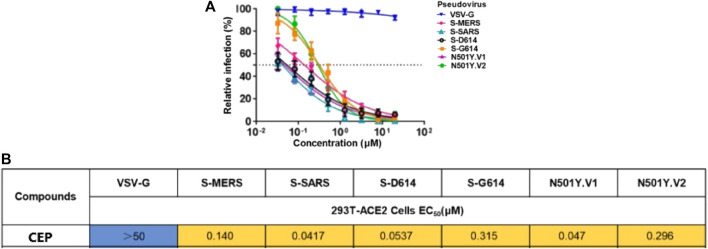
Dose–response curves of CEP against pseudoviruses (VSV-G, S-MERS, S-SARS, S-G614, N501Y.V1, and N501Y.V2) in the 293T-ACE2 cell line **(A)**; EC_50_ values of CEP against the entry of different pseudoviruses in the 293T-ACE2 cell line **(B)** (He et al., 2021).

**FIGURE 6:**
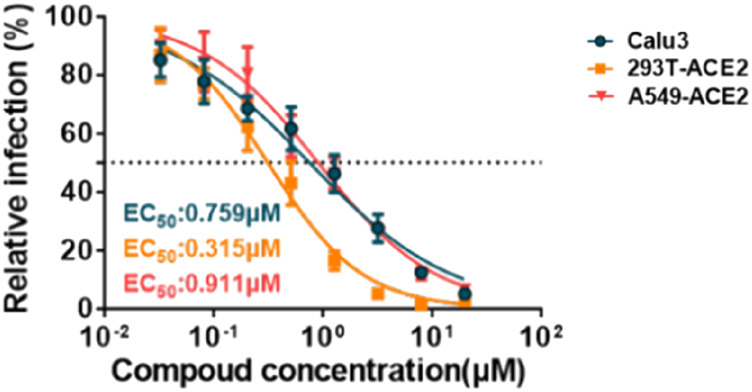
Efficacy of CEP in Calu-3, 293T-ACE2, and A549 cell lines against the S-G614 coronavirus (He et al., 2021).

In March 2021, Huang et al. from Chongqing Medical University in China reported a set of bisbenzylisoquinoline alkaloids (such as CEP, hernandezine, and tetrandrine) as pan-coronavirus entry inhibitors, of which CEP exhibited the highest efficacy. According to this study, CEP could usefully protect different cell lines (293T-ACE2, Calu-3, and A549) from infection by different coronaviruses (SARS-CoV, MERS-CoV, and SARS-CoV-2 [S-D614, S-G614, and N501Y variants]) *in vitro* (Table 2-E) (He et al., 2021). The dose–response curves of CEP against pseudoviruses (VSV-G, S-MERS, S-SARS, S-G614, N501Y.V1, and N501Y.V2) in the 293T-ACE2 cell line, the EC_50_ values of CEP against the entry of different pseudoviruses in the 293T-ACE2 cell line, and the efficacy of CEP in Calu3, 293T-ACE2, and A549 cell lines against S-G614 coronavirus are shown in Figures 5, 6. They further confirmed that CEP blocked host calcium channels, thus inhibiting Ca^2+^-mediated fusion and suppressing viral entry. In addition, CEP upregulated intracellular cholesterol levels, which might also help suppress viral infections (He et al., 2021).

In July 2021, Savaş Tay et al. from the University of Chicago published relevant data on “New Use of Old Drugs” in Science, confirming that CEP had the best *in vitro* inhibitory activity against SARS-CoV-2 among 1,900 clinically safe drugs. CEP demonstrated an EC_50_ value of only 0.13 μmol/L in A549-ACE2 cells, which was superior to that of remdesivir, with an EC_50_ value of 0.72 μmol/L (Table 2-F) (Drayman et al., 2021). In this study, 1,900 clinically safe drugs were screened *in vitro* using the human coronavirus OC43 model, and 108 drugs were finally identified that significantly reduce OC43 infection in A549 cells. Among these, only five drugs (elbasvir, amphotericin B, cediranib (AZD2171), CEP, and remdesivir) had an EC_50_ value of less than 1 μmol/L against OC43 virus (Drayman et al., 2021). Subsequently, the inhibitory activity of drugs that effectively inhibit OC43 was further verified against SARS-CoV-2 (Drayman et al., 2021). The results illustrated that CEP was the most efficient drug against SARS-CoV-2 at the cell level *in vitro* among these 1,900 clinically safe drugs (Drayman et al., 2021). Furthermore, this study found that CEP did not inhibit 3CL protein activity in 293T cells, indicating that the inhibitory activity of CEP against SARS-CoV-2 was not achieved by 3CL protein(Table 2-F) (Drayman et al., 2021). Interestingly, Javad Sharifi-Rad et al. from the University of Azuay, Ecuador, reported that CEP exhibited good binding affinities with the 3CL protein using molecular docking, which suggests that further studies are needed to investigate the mechanism of its anti-SARS-CoV-2 activity (Table 2-G) (Hossain et al., 2022).

**FIGURE 7:**
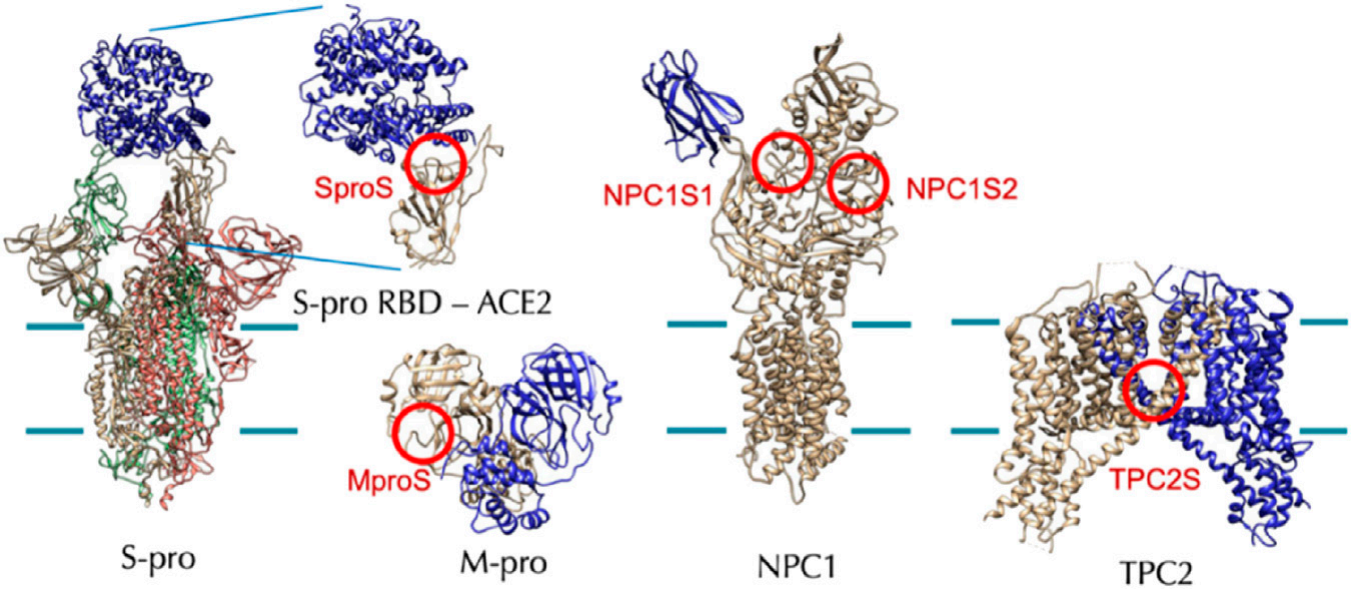
Hypothetical target proteins and target sites of CEP analogs (RBD: receptor-binding domain) (Hijikata et al., 2022).

In December 2021, Tsuyoshi Shirai et al. from Nagahama Institute of Bio-Science and Technology, Japan, conducted a study to investigate the anti-SARS-CoV-2 activity of 24 natural CEP analogs (Hijikata et al., 2022). They used molecular docking simulations to predict the binding affinities of these analogs, which were extracted from the KNApSAcK database (a comprehensive species–metabolite relationship database, http://kanaya.naist.jp/KNApSAcK/), to various target proteins including the spike protein, the main protease of SARS-CoV-2, NPC1, and TPC2 in humans (Hijikata et al., 2022). Hypothetical target proteins and target sites of CEP analogs simulated by molecular docking are shown in Figure 7. The target sites are indicated with red circles. The cell membrane boundaries are shown with green lines for the membrane proteins.

**FIGURE 8:**
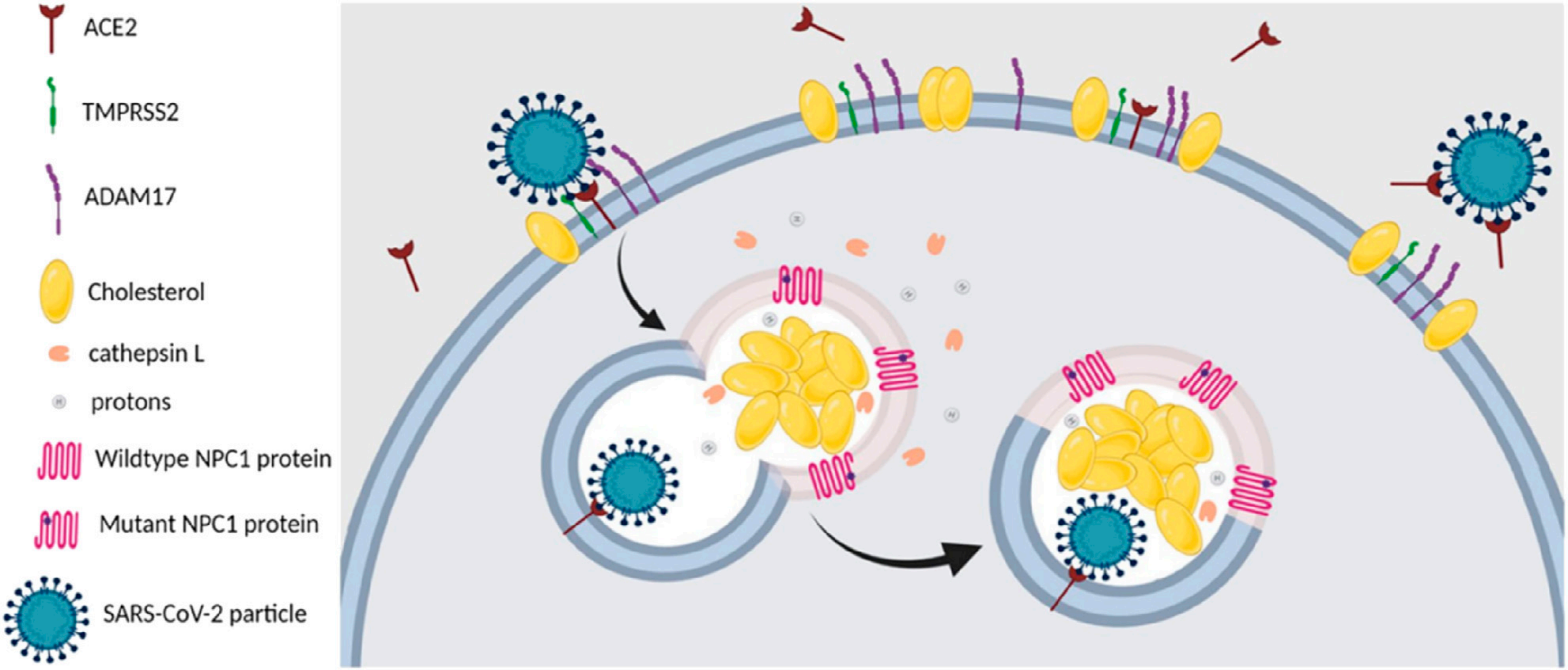
SARS-CoV-2 entry and infection in NPC1-deficient cells are negatively affected at several stages. First, NPC-related reduction in the number and cholesterol content of lipid rafts in the plasma membrane of NPC cells destabilizes ACE2 and TMPRSS2, both of which are located within these membrane domains. Second, the increase in plasma membrane levels of ADAM17 in NPC cells leads to increased shedding of ACE2, which hinders viral attachment and docking to host cells. Third, the abnormalities in the localization and activities of cathepsin L, induced by NPC1 deficiency, would reduce the likelihood of successful viral fusion after the endosome carrying the viral particle fuses with the NPC1-deficient lysosome. Fourth, the elevated levels of the antiviral oxysterols, 25-HC and 7-KC, in NPC cells would impede viral fusion and subsequent replication (Ballout et al., 2020).

CEP was found to physically interact with and inhibit the Niemann–Pick disease, type C1 (NPC1) protein, leading to lysosomal cholesterol accumulation and elevated intra-lysosomal pH, which resulted in a cellular phenocopy of NPC (Table 2-H) (Lyu et al., 2017). Therefore, it was feasible that the anti-SARS-CoV-2 capacity of CEP could be mediated, at least partially, by its lysosomotropic effects on the NPC1 protein of directly inhibiting the NPC1 protein and inducing a cellular phenocopy of NPC (Ballout et al., 2020). In summary, the NPC-related intracellular abnormalities induced by CEP may decrease the probability of successful entry, trafficking, and fusion of SARS-CoV-2 in NPC cells, as illustrated in Figure 8.

Researchers evaluated the anti-SARS-CoV-2 capacity of CEP and selected analogs using Vero E6/TMPRSS2 cell-based SARS-CoV-2 infection assays. The results documented that CEP was most effective in suppressing viral proliferation, with IC_50_ and IC_90_ values of 1.90 and 4.46 μmol/L, respectively (Table 2-H). This was much better than its analogs, such as tetrandrine, berbamine, dauricine, and liensinine (Hijikata et al., 2022). Additionally, this study suggested that the diphenyl ester moiety of the chemical structure could be a potential pharmacophore for CEP analogs (Hijikata et al., 2022).

In 2022, Ding et al. from Tsinghua University and the Chinese Academy of Medical Sciences & Peking Union Medical College in China utilized the ChIRP-MS method to identify host factors that interact with important RNA virus pathogens such as SARS-CoV-2, Zika, and Ebola viruses. They screened drugs that target virus–host factor interactions and found some common and virus-specific host responses and vRNA-associated proteins that separately regulate viral infection (Zhang et al., 2022). Their findings revealed that SARS-CoV-2 binds and hijacks the host factor IGF2BP1 to stabilize vRNA and enhance viral translation (Zhang et al., 2022). Their research also showed that CEP effectively inhibits the original strain of SARS-CoV-2 with ACE2 overexpressing A549 cell line infection, with an IC_50_ value of 1.67 μmol/L and a CC_50_ value of 30.92 μmol/L (Table 2-I). Additionally, CEP could efficiently restrain the infection of the SARS-CoV-2 variant (B.1.351) in A549 and Huh-7.5.1 cell lines with an IC_50_ value of 0.24 μmol/L and 0.06 μmol/L, respectively (Table 2-I). For Zika and Ebola viruses, CEP also demonstrated a powerful antiviral effect, with an IC_50_ value of 2.19 μmol/L in the Huh-7 cell line for Zika and 0.42 μmol/L in Huh-7.5.1-VP30 cells for Ebola (Zhang et al., 2022) (Table 2-I). CEP exhibited a strong binding force with phosphatidylinositol 3-kinase (PIK3) CA, PIK3CD, AKT serine/threonine kinase 1 (AKT1), and ACE2 and plays a significant role in the treatment of COVID-19 by regulating PI3K-Akt, relaxin, vascular endothelial growth factor (VEGF), and HIF-1 signaling pathways (Jiang et al., 2022). The researchers also estimated the anti-SARS-CoV-2 effect of CEP *in vivo* using hACE2 transgenic mice. About 10 mg/kg CEP was intranasally administered daily to mice. Compared with the control group, the viral load in the CEP group showed a significant reduction at 5 dpi, but no obvious differences at 3 dpi. Moreover, the expression levels of TNF and of IL6 were also reduced in the CEP group (Zhang et al., 2022). In the control group, SARS-CoV-2-infected mice showed inflammation in lung tissues, which contained a protein-rich fluid exudate. However, although some injuries and inflammation were observed in the CEP group, the extent of damage was much less severe in the lung tissue (Zhang et al., 2022). Moreover, further research revealed that the combination of CEP and trifluoperazine (5 µM each) reduced the viral RNA level to less than 0.01% in the Huh-7.5.1 cell line. This inhibition was approximately 50-fold stronger than that by CEP alone and 1,000-fold stronger than that by trifluoperazine alone (Zhang et al., 2022).

In July 2022, Tong et al. from Beijing University of Chemical Technology established a cellular model using the coronavirus GX_P2V infection of Vero E6 cells in July 2022 (An et al., 2022). They used N-glycoproteomic analysis to investigate the effect of coronavirus GX_P2V on host cell protein glycosylation and analyzed the antagonistic effect of CEP on abnormal protein glycosylation caused by coronavirus (An et al., 2022). The results revealed that GX_P2V could contribute to abnormal changes in protein glycosylation levels in host cells, while CEP could partially antagonize the abnormal protein glycosylation caused by GX_P2V(Table 2-A) (An et al., 2022). Additionally, CEP could regulate the glycosylation level of coronavirus S protein (An et al., 2022) (Table 2-A). Furthermore, Kumar et al. (2022) reported a rapid, stratified two-step screening approach using pseudovirus entry inhibition assays, followed by an infectious prototypic SARS-CoV-2 cytotoxic effect inhibition assay in multiple cell lines and subsequently screened a library of FDA-approved and clinical-stage drugs. They identified that CEP, berbamine, apilimod, and (S)-crizotinib could potently inhibit SARS CoV-2-induced cell death. These results suggest that CEP could exert anti-SARS-CoV-2 effects at a low concentration *in vitro* and *in vivo* with remarkable efficacy and safety, indicating that CEP has enormous potential clinical value in the treatment of COVID-19. However, one of the limitations of our review is that these modeling studies (such as HCoV-OC43 and GX_P2V models) are only predictive and not definitive proof that CEP engages these viral enzymes. Further research is needed to confirm these findings. Another limitation is that several of the cited studies in Table 2 did not report the CC_50_ value of CEP *in vitro*. Therefore, it is difficult to determine the specificity of the antiviral action independent of CEP-induced toxicity. Future studies should include both IC_50_ and CC_50_ measurements to provide a more comprehensive understanding of the antiviral potential and safety profile of CEP.

## 4 The pharmacokinetic effect of CEP

Pharmacokinetic studies have become increasingly important in drug discovery and development. Such studies help evaluate concentration–effect relationships, design novel drug delivery systems, and optimize drug dosing regimens, among other things (Cremers et al., 2020; Chen et al., 2022). Therefore, pharmacokinetic studies of CEP could provide valuable information for optimizing drug efficacy and studying toxicology, clinical pharmacology, therapeutics, and drug–drug interactions. For CEP, several methods for quantitative analysis have been established, including ultrahigh-performance liquid chromatography–mass spectroscopy (UHPLC-MS/MS) (Bixia et al., 2020; Gu et al., 2021; Li et al., 2022), high-performance liquid chromatography–mass spectroscopy (HPLC-MS/MS) (Hao et al., 2010; Dong et al., 2011; Guo et al., 2013; Deng et al., 2017), high-performance liquid chromatography-ultraviolet detection (HPLC-UV) (Moro et al., 1989; Xu et al., 2007; Desgrouas et al., 2014; Gao et al., 2021), and ultraviolet (UV) spectrophotometry (Moro et al., 1989). Actually, methods for the determination of CEP in rat and human plasma using HPLC-MS/MS, respectively, established and verified by Deng et al. (2017) and Guo et al. (2013) obtained the best sensitivity with the lower limit of quantification (LLOQ) at 0.1 ng/mL. To date, the pharmacokinetics of CEP has been evaluated in different dosages, routes, and species, including rats, mice, rabbits, dogs, and humans. The findings of these studies are summarized in Tables 3–5).

**TABLE 3:** Pharmacokinetic parameters of CEP in diverse dosages and routes of administration in rats.

N.O.	Drug	Species	Usage	Dosage (mg/kg)	Pharmacokinetic parameters		Bioavailability (F %)	Sample size	Detection method and LLOQ	References
A	CEP	Rat	i.v., single treatment in the tail lateral vein	1	C_max_ (ng/mL)	153.7 ± 16.18	—	*n* = 6	HPLC-MS/MS; 0.5 ng/mL	Deng et al. (2017)
					T_max_ (h)	—				
					t_1/2_ (h)	6.76 ± 1.21				
					AUC_(0–t)_ (ng/mL·h)	717.81 ± 158.35				
					AUC_(0–∞)_ (ng/mL·h)	721.80 ± 160.76				
					MRT_(0–t)_ (h)	7.04 ± 0.49				
					MRT_(0–∞)_ (h)	7.30 ± 0.51				
					Clz (L/h·kg)	1.431 ± 0.31				
					Vz (L/kg)	13.79 ± 1.76				
B	CEP		p.o., single treatment	10	C_max_ (ng/mL)	46.89 ± 5.25	5.65 ± 0.35	*n* = 6		
					T_max_ (h)	2.67 ± 1.16				
					t_1/2_ (h)	11.02 ± 1.32				
					AUC_(0–t)_ (ng/mL·h)	406.63 ± 62.57				
					AUC_(0–∞)_ (ng/mL·h)	422.26 ± 66.91				
					MRT_(0–t)_ (h)	10.49 ± 0.62				
					MRT_(0–∞)_ (h)	12.45 ± 1.20				
					Clz (L/h·kg)	24.08 ± 2.42				
					Vz (L/kg)	381.37 ± 61.63				
C	CEP	Rat	i.v., single treatment in the tail lateral vein	1	C_max_ (ng/mL)	148.8 ± 60.1	—	*n* = 4	UPLC-MS/MS; 5 ng/mL	Li et al. (2022)
					T_max_ (h)	—				
					t_1/2_ (h)	2.80 ± 0.42				
					AUC_(0–t)_ (ng/mL·h)	576.2 ± 114.1				
					MRT_(0–t)_ (h)	16.0 ± 1.8				
					Clz (L/h·kg)	1.74 ± 0.33				
					Vz (L/kg)	7.01 ± 1.69				
D	CEP		p.a., pulmonary drug delivery via the trachea using a nebulizer and laryngoscope, single treatment	1	C_max_ (ng/mL)	65.3 ± 16.1	68.07	*n* = 4		
					T_max_ (h)	0.017 ± 0.000				
					t_1/2_ (h)	16.35 ± 1.67				
					AUC_(0–t_) (ng/mL·h)	392.2 ± 43.7				
					MRT_(0–t)_ (h)	15.4 ± 1.0				
					Clz (L/h·kg)	2.22 ± 0.37				
					Vz (L/kg)	52.3 ± 9.6				
E	CEP		p.o., single treatment	10	C_max_ (ng/mL)	31.8 ± 14.6	13.15	*n* = 4		
					T_max_ (h)	13.50 ± 7.55				
					t_1/2_ (h)	17.15 ± 3.14				
					AUC_(0–t)_ (ng/mL·h)	757.8 ± 144.7				
					MRT_(0–t)_ (h)	20.7 ± 3.5				
					Clz (L/h·kg)	9.14 ± 2.92				
					Vz (L/kg)	218.0 ± 39.8				
F	CEP	Rat	p.a., single treatment	1	C_max_ (ng/mL)	65.27 ± 16.05	64.05	*n* = 3	UPLC-MS/MS; 5 ng/mL	Gu et al. (2021)
					T_max_ (h)	0.017 ± 0.000				
					t_1/2_ (h)	14.64 ± 2.05				
					AUC_(0–∞)_ (ng/mL·h)	382.7 ± 46.7				
					MRT_(0–∞)_ (h)	30.4 ± 5.8				
					ke(1/h)	0.04 ± 0.006				
G	CEP		i.v., single treatment in the tail lateral vein	1	C_max_ (ng/mL)	148.82 ± 60.08	—	*n* = 4		
					T_max_ (h)	—				
					t_1/2_ (h)	19.02 ± 4.46				
					AUC_(0–∞)_ (ng/mL·h)	597.4 ± 113.4				
					MRT_(0–∞)_ (h)	18.9 ± 2.4				
					ke(1/h)	0.038 ± 0.008				
H	CEP	Rat	p.o., single treatment	40	C_max_ (ng/mL)	430 ± 60	—	*n* = 6	HPLC-UV; 50 ng/mL	Gao et al. (2021)
					T_max_ (h)	4.25 ± 2.95				
					t_1/2_ (h)	4.86 ± 3.08				
					AUC_(0–∞)_ (ng/mL·h)	4,660 ± 1,490				
I	CEP-SEDDS		p.o., single treatment	40	C_max_ (ng/mL)	1,000 ± 480	203.64; relative bioavailability	*n* = 6		
					T_max_ (h)	2.92 ± 0.20				
					t_1/2_ (h)	8.65 ± 4.97				
					AUC_(0–∞)_ (ng/mL·h)	9,490 ± 1,680				

C_max_: peak plasma concentration; T_max_: the corresponding time taken to reach C_max_; t_1/2_: the terminal elimination half-life; MRT: mean residence time; AUC_0-t_/AUC_0-∞_: areas under the plasma concentration–time curve from time-zero to the last quantifiable time point and to infinity; CLz: clearance rate; Vz: apparent volume of distribution.

**TABLE 4:** Pharmacokinetic parameters of CEP in diverse dosages and route of administration in mice, rabbits, and dogs.

N.O.	Drug	Species	Usage	Dosage (mg/kg)	Pharmacokinetic parameters		Bioavailability (F %)	Sample size	Detection method and LLOQ	References
A	CEP	Mice	i.p., single treatment	21	C_max_ (ng/mL)	874	—	24	HPLC-UV; 75 ng/mL	Desgrouas et al. (2014)
					T_max_ (h)	0.25				
					t_1/2_ (h)	2.52				
					AUC_(0–6h)_ (ng/mL·h)	2,069				
					Clz (L/h·kg)	8.77				
					Vz (L/kg)	31.8				
B	CEP	Infected mice	i.p., single treatment	21	C_max_ (ng/mL)	**1.088 #**		24		
					T_max_ (h)	0.25				
					t_1/2_ (h)	2.59				
					AUC_(0–6h)_ (ng/mL·h)	2,205				
					Clz/F (L/h·kg)	8.04				
					Vd/F (L/kg)	30.1				
C	CEP	Dog	i.v., single treatment, administered by intravenous infusion using an infusion pump over 1 h	3	C_max_ (ng/mL)	262.6 ± 5.09	—	*n* = 5	HPLC-MS/MS; 5 ng/mL	Dong et al. (2011)
					T_max_ (h)	—				
					t_1/2_ (h)	14.55 ± 2.46				
					AUC_(0–t)_ (ng/mL·h)	1,376.85 ± 428.45				
					AUC_(0–∞)_ (ng/mL·h)	1,506.83 ± 434.82				
					MRT_(0–t)_ (h)	12.24 ± 1.98				
					Clz (L/h·kg)	2.14 ± 0.66				
					Vz (L/kg)	44.77 ± 5.76				
D	CEP	Rabbit	i.v., single treatment in the marginal ear vein	200	C_max_ (μg/mL)	0.49 ± 0.02	—	*n* = 4	Acid–base titration using 0.7 μg/mL	Li et al. (1997)
					T_max_ (h)	—				
					t_1/2_ (h)	t_1/2a_: 0.204 t_1/2β_: 4.62				
					AUC (μg/mL·h)	966.5				
					Cl (L/h·kg)	213.8				
					Vd (L/kg)	1,379				

**TABLE 5:** Pharmacokinetic parameters of CEP in different dosages and route of administration in humans.

N.O.	Drug	Usage	Dosage (mg)	Pharmacokinetic parameters		Bioavailability (F %)	Sample size	Detection method and LLOQ	References
A	CEP	p.o., single treatment	10	C_max_ (ng/mL)	0.53 ± 0.06	—	*n* = 2	HPLC	Kohtaro et al. (1989), Moro et al. (1989)
				T_max_ (h)	2.5 ± 0.5 1				
				t_1/2_ (h)	4.1 ± 0.1				
				AUC_(0–t)_ (ng/mL·h)	2.78 ± 0.13				
				AUC_(0–∞)_ (ng/mL·h)	3.49 ± 0.09				
				CL (L/h)	-				
B			30	C_max_ (ng/mL)	2.35 ± 0.48	—	*n* = 5		
				T_max_ (h)	1 ± 0.2				
				t_1/2_ (h)	9.2 ± 1.3				
				AUC_(0–t)_ (ng/mL·h)	18.6 ± 4.5				
				AUC_(0–∞)_ (ng/mL·h)	23.8 ± 6.1				
				CL (L/h)	1,792 ± 574				
C			60	C_max_ (ng/mL)	3.46 ± 0.27	—	*n* = 5		
				T_max_ (h)	1.1 ± 0.2				
				t_1/2_ (h)	6.8 ± 0.5				
				AUC_(0–t)_ (ng/mL·h)	27.4 ± 3.2				
				AUC_(0–∞)_ (ng/mL·h)	26.4 ± 2.8				
				CL (L/h)	2,390 ± 285				
D			120	C_max_ (ng/mL)	6.78 ± 1.11	—	*n* = 5		
				T_max_ (h)	1.2 ± 0.3				
				t_1/2_ (h)	t_1/2a_: 3.3 ± 1.0 t_1/2β_: 17.1 ± 4.1				
				AUC_(0–t)_ (ng/mL·h)	65.9 ± 7.4				
				AUC_(0–∞)_ (ng/mL·h)	131.3 ± 28.5				
				CL (L/h)	1,094 ± 228				
E		i.v., single treatment in 5 min	25	C_max_ (ng/mL)	187 ± 14	9	*n* = 5	HPLC	
				t_1/2_ (h)	35.8 ± 3.2				
				AUC_(0–48 h)_ (ng/mL⋅h)	110.4 ± 92				
				AUC_(0–∞)_ (ng/mL·h)	158.8 ± 15.8				
				CL (L/h)	164 ± 18				
F	CEP	i.v., single treatment in 5 min	50	C_max_ (ng/mL)	433 ± 25	6	*n* = 5		
				t_1/2_ (h)	36.9 ± 3.6				
				AUC_(0–48 h)_ (ng/mL⋅h)	252.9 ± 14.8				
				AUC_(0–∞)_ (ng/mL·h)	377.8 ± 22.7				
				CL(L/h)	136 ± 17				
G	CEP	i.v., single treatment in 5 min	100	C_max_ (ng/mL)	1,464 ± 364	9	*n* = 5		
				t_1/2_ (h)	31.8 ± 0.8				
				AUC_(0–48 h)_ (ng/mL⋅h)	730.5 ± 86.2				
				AUC_(0–∞)_ (ng/mL·h)	962.5 ± 101.6				
				CL(L/h)	102 ± 8				
H	CEP	i.v., treatment in 5 min, continuously for 7 days	100	C_max_ (ng/mL)	196.3 ± 27.50	—	*n* = 5		
				C_min_ (ng/mL)	34.0 ± 2.32				
				t_1/2_ (h)	62.0 ± 2.8				
I	CEP	i.v., single treatment in 60 min	50	C_max_ (ng/mL)	135.9 ± 66.9	—	*n* = 12	HPLC-MS/MS;0.1 ng/mL	Hao et al. (2010)
				T_max_(h)	0.75 ± 0.21				
				t_1/2_ (h)	131.9 ± 48.4				
				AUC_(0–192 h)_ (ng/mL·h)	566.6 ± 216.6				

### 4.1 The pharmacokinetic effect of CEP in rats

Deng et al. (2017) investigated the pharmacokinetic characteristics of CEP in Sprague–Dawley (SD) rats after intravenous and oral administration using a sensitive HPLC-MS/MS method. The calibration curve was linear within the range of 0.1–200 ng/mL (*r* ^2^ = 0.999) with an LLOQ of 0.1 ng/mL. The study involved 12 SD rats, randomized equally into an intravenous group (1 mg/kg, n = 6) and an orally treated group (10 mg/kg, n = 6).

Following a single treatment of CEP (1 mg/kg) via the tail lateral vein, the peak concentration (C_max_), half-life (t_1/2_), area under the concentration/time curve (AUC_0-t_), mean residence time (MRT_0-t_), clearance rate (CLz), and apparent volume of distribution (Vz) were determined to be 153.7 ± 16.18 ng/mL, 6.76 ± 1.21 h, 717.81 ± 158.35 ng/mL·h, 7.04 ± 0.49 h, 1.431 ± 0.31 L/kg·h, and 13.79 ± 1.76 L/kg, respectively (Table 3-A) (Deng et al., 2017). When CEP was orally administered at a single dose of 10 mg/kg to rats, the mean values of C_max_, T_max_, t_1/2_, AUC_0-t_, MRT_0-t_, CLz, and Vz were 46.89 ± 5.25 ng/mL, 2.67 ± 1.16 h, 11.02 ± 1.32 h, 406.63 ± 62.57 ng/mL·h, 10.49 ± 0.62 h, 24.08 ± 2.42 L/kg·h, and 381.37 ± 61.63 L/kg, respectively (Table 3-B) (Deng et al., 2017). These results indicate that CEP is not readily absorbed and is slowly distributed and eliminated in SD rats (Deng et al., 2017). The absolute bioavailability of CEP via oral delivery was found to be 5.65% ± 0.35% (Table 3-B) (Deng et al., 2017), indicating that CEP has poor absorption in SD rats through oral administration. These findings suggest that further research is needed to examine the factors contributing to the poor bioavailability of CEP and to develop strategies to improve it.

Li et al. (2022) improved the bioavailability of CEP by optimizing its solubility and through pulmonary delivery. They established and validated a UPLC-MS/MS method for determination of CEP in SD rat plasma to support research on bioavailability and pharmacokinetics. The calibration curve of CEP was linear in the concentration range of 0.5–100 ng/mL (*r* ^2^ > 0.997), with the LLOQ at 0.5 ng/mL. They tested different conditions to improve the poor solubility of CEP in water and found that an acidic vehicle was the key factor for improving CEP dissolution. Solutions of CEP with concentrations of 2.0 and 30 mg/mL were prepared in pH 3.7 and pH 2.8 acidic saline regulated by acetic acid, respectively, in which CEP powder was fully dissolved by vortexing for 20 s (Li et al., 2022). Twelve SD rats were equally randomized into intravenous (i.v., *n* = 4), pulmonary (p.a., *n* = 4), and oral administration (p.o., *n* = 4) groups at a single dose of 1, 1, and 10 mg/kg, respectively. After a single intravenous or pulmonary administration of 1 mg/kg, CEP reached its C_max_ in the plasma directly at the first sampling time with mean C_max_ values of 148.8 ± 60.1 ng/mL and 65.3 ± 16.1 ng/mL, t_1/2_ of 2.80 ± 0.42 h and 16.35 ± 1.67 h, and AUC_0-t_ of 576.2 ± 114.1 ng/mL·h and 392.2 ± 43.7 ng/mL·h (Table 3-C, D) (Li et al., 2022). After a single oral administration of 10 mg/kg, the mean C_max_, T_max_, t_1/2_, and AUC_0-t_ were 31.8 ± 14.6 ng/mL, 13.50 ± 7.55 h, 17.15 ± 3.14 h, and 757.8 ± 144.7 ng/mL·h, respectively (Table 3-E) (Li et al., 2022). The absolute bioavailability of CEP through pulmonary administration was 68.07% (Table 3-D), which improved over five-fold compared to oral administration (13.15%, Table 3-E) (Li et al., 2022). Interestingly, in this study, the t_1/2_ of CEP in intravenous, pulmonary, and oral administration was 2.80 ± 0.42 h, 16.35 ± 1.67 h, and 17.15 ± 3.14 h, respectively. This means that the t_1/2_ of CEP in pulmonary and oral administration was over 5.8 and 6.1 times higher than in intravenous administration, respectively, seemingly indicating that CEP was eliminated much more quickly when administered intravenously. Meanwhile, Gu et al. (2021) also confirmed that the absolute bioavailability of CEP could be significantly improved through pulmonary administration via the trachea using a nebulizer and laryngoscope. They developed a sensitive and rapid UPLC-MS/MS method for the determination of CEP in SD rat plasma. CEP solution with the concentration of 5.0 ng/mL was prepared with pH 3.5 acetic acid (Gu et al., 2021). Seven SD rats were randomized into intravenous (i.v., *n* = 4) and pulmonary (p.a., *n* = 3) groups at a single dose of 1 mg/kg. After a single intravenous or pulmonary administration of 1 mg/kg, CEP reached its C_max_ in the plasma instantly at the first sampling time with mean C_max_ values of 148.82 ± 60.08 ng/mL and 65.27 ± 16.05 ng/mL, t_1/2_ of 19.02 ± 4.46 and 14.64 ± 2.05 h, AUC_0-**∞**_ of 597.4 ± 133.4 ng/mL·h and 382.7 ± 46.7 ng/mL·h, and MRT_0-t_ of 18.9 ± 2.4 h and 30.4 ± 5.8 h, respectively (Table 3-F, G) (Gu et al., 2021). The t_1/2_ of CEP in intravenous and pulmonary administration was roughly equal (19.02 ± 4.46 h vs. 14.64 ± 2.05 h). Compared with Li et al. (2022)’sresearch, as described previously (the t_1/2_ of CEP in pulmonary administration was over 5.8 times higher than that in intravenous administration), it is confusing in a way. Anyway, these results confirmed that the absolute bioavailability of CEP could be significantly improved through pulmonary administration via the trachea using a nebulizer and laryngoscope, which indicated that aerosol inhalation of CEP might be a potential method. However, the limitation was the minor sample size (n ≤ 4) of these two studies.

Gao et al. (2021) developed a self-emulsifying drug delivery system (SEDDS) loaded with CEP to improve its oral bioavailability in rats. Isopropyl palmitate (IPP), Cremophor RH40, and Macrogol 200 (PEG 200) were chosen as the oil phase, emulsifier, and co-emulsifier, respectively. The optimized condition was CEP: IPP: Cremophor RH40: PEG 200 = 3.6:30.0:55.3:11.1 in the mass ratio, with a maximum drug loading of 36.21 mg/mL (Gao et al., 2021). The samples were analyzed using the HPLC system equipped with a UV detector, set at 235 nm. The calibration curve of CEP was linear from 0.05 to 1.6 μg/mL, with a correlation coefficient of 0.9985 (Gao et al., 2021). Twelve male SD rats were randomly assigned to two groups (*n* = 6). After a single oral gavage administration of 40 mg/kg of CEP-SEDDS or CEP suspension, CEP reached its C_max_ values of 1.00 ± 0.48 μg/mL or 0.43 ± 0.06 μg/mL, separately, whereas the T_max_ value was 2.92 ± 0.20 h or 4.25 ± 2.95 h, and t_1/2_ was 8.65 ± 4.97 h or 4.86 ± 3.08 h, respectively (Table 3-H, I) (Gao et al., 2021). The AUC_0-t_ of CEP-SEDDS and CEP was 9.49 ± 1.68 μg/mL·h and 4.66 ± 1.49 μg/mL·h, respectively (Table 3-H, I) (Gao et al., 2021). Compared with the CEP suspension, the single oral gavage administration of CEP-SEDDS at the dosage of 40 mg/kg showed over two-fold increase in the AUC_0-t_ of CEP, and the relative bioavailability of CEP-SEDDS was 203.64% (Table 3-I), indicating that the oral bioavailability of CEP was improved after it was prepared into SEDDS.

### 4.2 The pharmacokinetic effect of CEP in mice, rabbits, and dogs

Desgrouas et al. (2014) established and validated a quantitative analysis of CEP in plasma by semi-automatic microextraction using a packed sorbent, combined with liquid chromatography. The LLOQ was 75 ng/mL, and the method was successfully used to determine the pharmacokinetic profile of CEP in healthy and *Plasmodium berghei*-infected BALB/c female mice. Healthy mice (*n* = 24) and infected mice (*n* = 24) received intraperitoneal (i.p.) administration of CEP at a dose of 21 mg/kg. The mean values of C_max_, T_max_, t_1/2_, AUC_0-t_, CL/F, and Vd/F of CEP in healthy mice were 874 ng/mL, 0.25 h, 2.52 h, 2,069 ng/mL·h, 8.77 L/h·kg, and 31.8 L/kg, respectively (Table 4-A) (Desgrouas et al., 2014). Correspondingly, the mean values of C_max_, T_max_, t_1/2_, AUC_0-t_, CL/F, and Vd/F of CEP in infected mice were 1.088 ng/mL, 0.25 h, 2.59 h, 2,205 ng/mL·h, 8.04 L/h·kg, and 30.1 L/kg, respectively (Table 4-B) (Desgrouas et al., 2014). According to these data, the mean C_max_ value in healthy mice was over 800 times higher than that in infected mice. Nevertheless, authors described that the pharmacokinetic parameters of CEP did not seem to be impacted by the infection (Desgrouas et al., 2014). In our opinion, there might be a few mistakes or blemishes in this article. For instance, the mean C_max_ value of CEP in infected mice might not be 1.088 ng/mL, but rather 1,088 ng/mL with a high probability. Furthermore, previous studies had confirmed that dogs have similar gastrointestinal physiology and anatomy to humans (Kararli, 1995; He et al., 2022). As a consequence, dogs were frequently used for safety evaluations and pharmacokinetic studies. Dong et al. (2011) developed and validated an HPLC-MS/MS method with the LLOQ at 5 ng/mL for the bioanalysis of CEP and applied it to its pharmacokinetic study in beagle dogs. They administered a single intravenous dose of 3 mg/kg CEP using an infusion pump over 1 h to adult male beagle dogs (*n* = 5) and found the corresponding values of C_max_, t_1/2_, AUC_0-t_, AUC_0-∞_, MRT_0-t_, CLz, and Vz to be 262.6 ± 5.09 ng/mL, 14.55 ± 2.46 h, 1,376.85 ± 428.45 ng/mL·h, 1,506.83 ± 434.82 ng/mL·h, 12.24 ± 1.98 h, 2.14 ± 0.66 L/h·kg, and 44.77 ± 5.76 L/kg, respectively (Table 4-C).

Rabbits are commonly used as animal models in pharmacokinetic studies (Huang et al., 2021). Li et al. (1997) intravenously injected CEP into the marginal ear vein of rabbits (*n* = 4) at a dosage of 200 mg/kg. The plasma concentration of CEP in whole blood decreased rapidly from 0.49 ± 0.02 μg/mL immediately after injection, and the decrease rate slowed down after 2.25 h, reaching equilibrium between 24 and 72 h. The concentration–time curve of CEP in rabbit blood was consistent with that of a two-compartment open model. The values for t_1/2a_ and t_1/2β_ were 0.204 h and 4.62 h, respectively (Li et al., 1997). The V_d_ was as high as 1,379 L/kg. The AUC and CL were 966.5 μg/mL·h and 213.8 L/h·kg, respectively (Table 4-D). The results illustrate that CEP is quickly distributed from the central compartment to the peripheral compartment, and the rate of CEP reentry from the peripheral compartment to the central compartment is slower. This indicated that CEP is rapidly distributed to tissues after intravenous injection and subsequently eliminated from the body. However, it should be noted that the sample size was quite small (*n* = 4), and pharmacokinetic parameters in this study were reported as means without corresponding standard deviations.

### 4.3 The pharmacokinetic effect of CEP in humans

As early as 1989, K. Yasuda et al. (Kohtaro et al., 1989; Moro et al., 1989) carried out the phase I clinical study of CEP in healthy humans based on the HPLC method and the one-compartment open model. The pharmacokinetics of CEP was investigated in healthy humans following single oral doses of 10 mg (*n* = 2), 30 mg (*n* = 5), 60 mg (n = 5), and 120 mg (*n* = 5), intravenously injected (single treatment in 5 min) at the doses 25 mg (*n* = 5), 50 mg (*n* = 5), and 100 mg (*n* = 5), and continuous intravascular administration (single treatment in 5 min for each time) at doses of 100 mg/day (*n* = 5) for 7 days, respectively (Kohtaro et al., 1989). The results demonstrated that the mean C_max_ or the AUC of CEP at the dosage from 10 to 120 mg showed linear relationships after oral administration, i.e., 0.5 ± 0.06 ng/mL, 2.35 ± 0.48 ng/mL, 3.46 ± 0.27 ng/mL, and 6.78 ± 1.11 ng/mL for C_max_ or 3.49 ± 0.09 ng/mL·h, 23.8 ± 6.1 ng/mL·h, 26.4 ± 2.8 ng/mL·h, and 131.3 ± 28.5 ng/mL·h for AUC_0-∞_ (Table 5-A–D). The T_max_ of CEP at the dosage of 10 to 120 mg ranged from 1.1 to 2.5 h (Table 5-A–D). What is worth mentioning is that the t_1/2_ ranged from 4.1–9.2 h at the dosage of 10 to 60 mg (Table 5-A–C). However, when the dosage increased to 120 mg, the disposition of CEP was consistent with that in the two-compartment model. Corresponding, t_1/2α_ and t_1/2β_ were 3.3 ± 1.0 h and 17.1 ± 4.1 h, respectively (Table 5-D). When CEP was administered in single intravenous doses in healthy subjects, linear relationships of the C_max_ or AUC were obtained among the dosages of 25, 50, and 100 mg (Table 5-E–G). The t_1/2_ ranged from 31.8–36.9 h, and the absolute bioavailability of CEP was from 6% to 9% (Table 5-E–G). The relationship between the C_max_ and AUC of oral administration and the dosage was significant and followed a linear relation, while the intravenous injection of CEP at a high dosage, such as 100 mg, showed a trend of saturation (Kohtaro et al., 1989; Moro et al., 1989). This might be due to the difference in the order of C_max_ and AUC_0-∞_, which has an obvious difference of more than 10 times between oral administration and intravenous injection. Intravenous injection at a high dosage, such as 100 mg, might result in the saturation of tissue compatibility, blood cell uptake, and liver metabolism (Moro et al., 1989). When CEP was intravenously injected in healthy subjects following 7 days of repeated doses of 100 mg/day, the steady-state concentration was obtained for five or six repeated doses of 100 mg/day approximately (Moro et al., 1989). Following the seven repeated doses, the peak (C_max_) and trough (C_min_) concentrations of CEP were 196.3 ± 26.50 ng/mL and 34.0 ± 2.32 ng/mL, respectively, and the t_1/2_ was 62.0 ± 2.8 h (Table 5-H). This t_1/2_ was about twice as long as that observed after a single intravenous administration (Moro et al., 1989). In 2010, Hao et al. (2010) described a rapid, sensitive, and specific method for determining CEP in human plasma by HPLC-MS/MS. This method was successfully applied to a pharmacokinetic study in which plasma concentrations of CEP in 12 healthy Chinese subjects were detected up to 192 h after a single intravenous administration of 50 mg CEP within 60 min. The calibration curve of CEP was linear from 0.5 to 200.0 ng/mL (*r* ^2^ = 0.9994) with the LLOQ at 0.5 ng/mL (Hao et al., 2010). The mean C_max_ of CEP was 135.9 ± 66.9 ng/mL, observed around 0.75 ± 0.21 h post-dosing. The t_1/2_ and AUC_0–192 h_ were 131.9 ± 48.4 h and 566.6 ± 216.6 ng/mL·h, respectively (Table 5-H) (Hao et al., 2010). Obviously, the mean C_max_ of CEP in this study was much lower than that reported by K. Yasuda et al. (Kohtaro et al., 1989; Moro et al., 1989) (433 ± 25 ng/mL), who administered the same dosage (50 mg/kg) intravenously. This difference might be explained by the difference in infusion duration, which was 60 min in this study instead of 5 min as reported in a previous study (Moro et al., 1989; Hao et al., 2010). Additionally, the t_1/2_ was intensely longer in this study (131.9 ± 48.4 h) than that reported by Yasuda et al. (Kohtaro et al., 1989; Moro et al., 1989) (36.9 ± 3.6 h). This might be relevant to the difference in the duration of sampling times and the LLOQ of CEP in plasma.

The sampling time was 192 h in this study, while in the former study, it was merely 48 h after dosing (Moro et al., 1989; Hao et al., 2010). Furthermore, the plasma concentration was determined by HPLC-MS/MS, with the LLOQ of CEP as low as 0.5 ng/mL, which was significantly lower than that in the previous study (Moro et al., 1989; Hao et al., 2010). Additionally, plasma concentrations of CEP fluctuated in the range of 1.2–2.6 ng/mL from 24 to 192 h after dosing (Hao et al., 2010), indicating that CEP was rapidly distributed into organs and mainly taken up by tissues, which strongly bound to CEP in the first 2 h after single intravenous administration and gradually eliminated *in vivo*, contributing to the fairly longer t_1/2_ (Moro et al., 1989; Hao et al., 2010).

### 4.4 The absorption and disposition of CEP

Previous studies have demonstrated that CEP is poorly absorbed and has low absolute bioavailability in rats, mice, rabbits, dogs, and human when administered orally (Kohtaro et al., 1989; Moro et al., 1989; Luo and Xia, 1993; Li et al., 1997; Hao et al., 2010; Dong et al., 2011; Desgrouas et al., 2014; Deng et al., 2017; Li et al., 2022). It is worth noting that in 2020, Bixia et al. (2020) used the UPLC-MS/MS method to determine the pharmacokinetics of CEP in rats and reported an absolute bioavailability of more than 41%, which differed significantly from all other study results (less than 13.15%) on the absolute bioavailability of CEP, as described previously (Moro et al., 1989; Deng et al., 2017; Li et al., 2022). This prompted that further research is needed, including more standardized and larger sample size pharmacokinetic or bioavailability studies.

Xu et al. (2007) inspected the absorption kinetics of CEP in the intestines of rats and the influence of different drug solution concentrations on absorption. The results revealed that CEP was well-absorbed in all segments of the intestine, mainly via a passive transport mechanism (Xu et al., 2007). The absorption percentage was over 15.4% in each segment (i.e., duodenum, jejunum, ileum, and colon), and no specific absorption was found in different segments (Xu et al., 2007). The absorption rate constant (ka) in the intestine did not significantly differ with CEP concentration (*p* > 0.05) (Xu et al., 2007). Hence, it was necessary to consider preparing oral sustained-release formulations of CEP to enhance its bioavailability.

YAMAKAWA et al. (1987) conducted a tissue distribution study examining the distribution of CEP in rats after intravenous administration using autoradiography and immunohistochemical techniques. The results showed that CEP distribution was high in the spleen, while in endocrine organs, such as the liver, kidney, lung, and gastrointestinal tract, the distribution was moderate (YAMAKAWA et al., 1987). Additionally, CEP was located in the heart, skeletal muscle, bone marrow, and lymphoid tissues when in lower concentrations (YAMAKAWA et al., 1987).

Furthermore, CEP was frequently distributed in matured T cells in the thymus (YAMAKAWA et al., 1987). Moreover, the study confirmed that CEP was excreted mainly through the gastrointestinal tract and partly though bile, urine, and expired air (YAMAKAWA et al., 1987). In another tissue distribution study, Luo and Xia (1993) randomly divided 50 rats into 10 groups (n = 5) and determined the concentration of CEP in the brain, heart, lung, liver, kidney, spleen, testis, and blood at 15 min, 30 min, 45 min, 1 h, 2 h, 4 h, 8 h, 12 h, 24 h, and 48 h after intragastric administration of CEP at 80 mg/kg. The results showed that CEP was rapidly and widely distributed in the tissues, with the highest concentration of CEP observed in the lungs (337.43 ± 28.65 μg/mL), followed by the liver (209.93 ± 9.33 μg/mL), spleen (181.0 ± 15.99 μg/mL), kidney (117.40 ± 14.58 μg/mL), heart (25.31 ± 2.72 μg/mL), brain (6.48 ± 0.66 μg/mL μg/mL), blood (1.92 ± 0.33 μg/mL), and testis (1.50 ± 0.48 μg/mL) (Luo and Xia, 1993). It is worth noting that the prior research had described the highest concentration of CEP in the spleen (YAMAKAWA et al., 1987), while in this study, the highest concentration of CEP was obtained in the lungs. The study also showed that the higher concentration of CEP in the brain tissue, than that in blood, indicated that it could pass through the blood–brain barrier (BBB). The highest concentration of CEP in the lung tissue, combined with its significant anti-SARS-CoV-2 capacity, highlights its enormous potential in COVID-19 treatment.

Zhang et al. (2020) investigated the effects of CEP on human liver CYP 1A2, 3A4, 2A6, 2E1, 2D6, 2C9, 2C19, and 2C8 *in vitro* using human liver microsomes (HLMs) with specific probe actions and probe substrates. They found that only the activity of CYP3A4, CYP2E1, and CYP2C9 was inhibited by CEP, with IC_50_ values of 16.29, 25.62, and 24.57 μmol/L, respectively. Enzyme kinetic studies showed that CEP was not only a noncompetitive inhibitor of CYP3A4 but also a competitive inhibitor of CYP2E1 and CYP2C9, with inhibition constant (Ki) values of 8.12, 11.78, and 13.06 μmol/L, respectively (Zhang et al., 2020). Therefore, in order to avoid or reduce the risk of adverse drug–drug interactions, CEP should be used with caution when co-administered with other drugs metabolized by CYP2E1 and CYP2C9, especially when administered intravenously.

## 5 Summary and discussion

CEP is a naturally occurring BBIQ alkaloid mainly derived from plants of the genus Stephania (Menispermaceae). It has been used as a safe, well-tolerated, and inexpensive drug since 1951 to treat many acute and chronic diseases, such as leukopenia (Nomoto et al., 2004; Bailly, 2019; Rogosnitzky et al., 2020), alopecia (Bailly, 2019; Rogosnitzky et al., 2020), malaria (Bailly, 2019), exudative middle-ear catarrh (Rogosnitzky and Danks, 2011; Rogosnitzky et al., 2020), idiopathic thrombocytopenic purpura (Nakayama et al., 1992; Rogosnitzky et al., 2020), and snake bites (Rogosnitzky and Danks, 2011; Bailly, 2019; Rogosnitzky et al., 2020). Additionally, it has been demonstrated to have significant antiviral effects against quite a few viruses, such as HIV-1 (Baba, 1997; Okamoto et al., 1998; Baba et al., 2001; Okamoto et al., 2001), HBV (Rogosnitzky et al., 2020), HSV-1 (Liu et al., 2004; Liu et al., 2021a; Liu et al., 2021b), HTLV (Toyama et al., 2012), ZikaV (Zhang et al., 2022), EboV (Zhang et al., 2022), PRRSV (Yang et al., 2021), PCV2 (Xu et al., 2020), SARS-CoV-2 (Zhang et al., 2005; Rogosnitzky et al., 2020; Wang and Yang, 2020), and HCOV-OC43 viruses (Kim et al., 2019). Recent studies have verified its remarkable antiviral capacity against SARS-CoV-2, both *in vitro* and *in vivo* (Lan et al., 2020; Rogosnitzky et al., 2020; White et al., 2020; He et al., 2021; Li et al., 2021; Ohashi et al., 2021; Fan et al., 2022; He et al., 2022; Hijikata et al., 2022; Hossain et al., 2022; Kumar et al., 2022; Li et al., 2022; Zhang et al., 2022). Monotherapy with CEP has been confirmed to inhibit viral entry and post-entry steps and attenuate the potential inflammatory effects that may result from viral infection. Better results (synergistic properties) could be obtained when combined with other antiviral medications such as NFV and lopinavir. Moreover, its low IC_50_/EC_50_ and CC_50_ and synergistic properties when combined with other antiviral medications establish its importance as a candidate in treating COVID-19.

It is necessary to calculate the ratio of C_max_ *in vivo*/IC_50_ *in vitro* to ensure whether the concentrations of the cellular effects of CEP are kept in perspective with the antiviral concentrations and what is actually achievable in humans. The IC_50_ values of CEP were different for various virus strains infecting different cell lines. The lowest IC_50_ value in the available data so far was 28.51 ng/mL (0.047 μmol/L), which occurred in the 293T-ACE2 cell line infected with SARS-CoV-2 N501Y.V1 (B.1.1.7). We used this IC_50_ (28.51 ng/mL) as a reference standard to calculate the ratio of C_max_ *in vivo*/IC_50_ *in vitro*, as shown in Table 6.

**TABLE 6:** Ratio of C_max_ of CEP in humans/IC_50_ of CEP for anti-SARS-COV-2 *in vitro*.

N.O.	Drug	Usage	Dosage (mg)	Cmax in humans (ng/mL)	IC_50_ *in vitro* (ng/mL)	C_max_/IC_50_ ratio	References
A	CEP	p.o.	10	0.53 ± 0.06	28.51	0.02	Kohtaro et al. (1989), Moro et al. (1989), Hao et al. (2010), He et al. (2021)
B			30	2.35 ± 0.48		0.08	
C			60	3.46 ± 0.27		0.12	
D			120	6.78 ± 1.11		0.24	
E		i.v., treatment in 5 min	25	187 ± 14		6.56	
F			50	433 ± 25		15.19	
G			100	1,464 ± 364		51.35	
H		i.v., treatment in 5 min, for 7 days	100	C_max:_ 196.3 ± 27.50		6.89	
				C_min:_ 34.0 ± 2.32		1.19	
I		i.v., treatment in 60 min	50	135.9 ± 66.9		4.77	

According to Table 6, a fairly high C_max_ of CEP can be achieved with i.v. administration, particularly the trough concentration and peak concentration of steady-state blood drug concentration after 7 consecutive days/IC_50_, which are 1.19 and 6.89, respectively, indicating that effective antiviral concentrations can be achieved in humans. However, due to the poor bioavailability of CEP *in vivo*, the ratio of C_max_/IC_50_ was only 0.24 when administering CEP at a single oral dose of 120 mg. This implies that further research is needed concerning dosage form modification or route of administration to increase the bioavailability of CEP. Additionally, by optimizing the structure of the lead compound CEP and changing its main metabolic pathway, the metabolic stability of the compound can be effectively improved, the action time of the drug *in vivo* can be prolonged, the exposure *in vivo* can be increased, the bioavailability can be improved, and the pharmacokinetic characteristics can be optimized.

Although fairly high C_max_ of CEP could be achieved with i.v. administration, an i.v. treatment is not a therapy that can be globally distributed and accessible. However, unfortunately, when CEP is administered at a conventional oral dose, the C_max_ of CEP in humans is so low that it is far from being close to the *in vitro* IC_50_ value for blocking SARS-CoV-2. Pharmacokinetic studies in animals confirmed that the absolute bioavailability of CEP obtained obviously improved (achieved over 64%) by pulmonary administration (shown in Table 3-D–F). So, pulmonary administration of CEP (such as atomization preparation, nasal spray, and powder for inhalation) might be a good way to accelerate the clinical application of CEP in the treatment of COVID-19.

Gao et al. (2021) utilized an SEDDS containing isopropyl palmitate, Cremophor RH40, and Macrogol 200 to enhance the oral bioavailability of CEP in rats. The optimal mass ratio was determined to be CEP: IPP: Cremophor RH40: PEG 200 = 3.6:30.0:55.3:11.1, with a maximum drug loading of 36.21 mg/mL. In a single oral gavage administration of 40 mg/kg of CEP-SEDDS or CEP suspension, CEP-SEDDS showed over two-fold increase in the AUC0-t of CEP and a relative bioavailability of 203.64% compared to CEP suspension, indicating improved oral bioavailability of CEP when formulated as SEDDS. These results demonstrate the potential of nanotechnology to improve the *in vivo* absorption and bioavailability of CEP, suggesting a need for further research.

It is important to consider whether CEP is a pan-assay interference compound (PAINS), given its bioactivity in so many *in vitro* and *in vivo* models. In recent years, the PAINS hypothesis has been widely used in the early screening of new drugs (Baell and Holloway, 2010; Pouliot and Jeanmart, 2016; Nelson et al., 2017; Adnan et al., 2022; Salin et al., 2022; Das et al., 2023). For example, curcumin has been classified as a typical PAINS compound due to its invalid metabolic panacea properties as its clinical studies have not provided strong evidence for a therapeutic effect (Nelson et al., 2017). To avoid the cost and input of ineffective compounds, Baell et al. proposed a set of PAINS screening compounds in 2010 (Baell and Holloway, 2010). They designed, constructed, and fully disclosed a database for a PAINS filter (PAINS-Remover, http://cbligand.org/PAINS/) to remove PAINS from screening libraries and exclude them from bioassays (Baell and Holloway, 2010). This PAINS filter has been widely recognized in the industry (Baell and Holloway, 2010; Pouliot and Jeanmart, 2016; Adnan et al., 2022; Salin et al., 2022; Das et al., 2023). We have screened the possibility of CEP being a PAINS compound. Fortunately, CEP has passed the PAINS filter, suggesting that it may not yield a false positive result due to interferences such as aggregating compounds, covalent bonding, and chelate formation when tested *in vitro*.

Drug-induced phospholipidosis can confound drug repurposing screens for SARS-CoV-2 as drugs that are active due to phospholipidosis are unlikely to translate *in vivo* (Tummino et al., 2021). Drug-induced phospholipidosis is the excessive deposition of drugs and phospholipids in cellular lysosomes (Chatman et al., 2009). Early detection of phospholipidosis could eliminate these artifacts, enabling a focus on molecules with therapeutic potential (Tummino et al., 2021). In fact, most of the small-molecule compounds that can induce phospholipidosis in cells and organs are cationic amphiphilic drugs (CADs), which are easily permeable to the lipid membrane (Chatman et al., 2009; Tummino et al., 2021). Although CEP is not a CAD, it is still necessary to counterscreen whether CEP can induce phospholipidosis in simple cellular assays (Morelli et al., 2006; Ceccarelli et al., 2017; Breiden and Sandhoff, 2019). This would allow investigators to focus on drugs with significant potential as antivirals.

Although a Canadian pharmaceutical company named PharmaDrug obtained a United States Patent (USP) for the enteric-coated formulation of CEP (PD-001, US10576007B2), which showed good absorbance and remarkably improved bioavailability, and submitted a phase I/II clinical trial application for PD-001 to the FDA to treat the SARS-CoV-2 infection and obtained an agreement on the development of PD-001 toward clinical studies for mild-to-moderate COVID-19 (Bauta et al., 2020; BioSpace, 2021), specific clinical trials for PD-001 have not yet been carried out. At present, no specific information about this clinical trial has been retrieved. Currently, the only registered clinical trial of CEP in the ClinicalTrials.gov database is the study of oral high-/low-dose cepharanthine compared With placebo in nonhospitalized adults with COVID-19 (ClinicalTrials.gov Identifier: NCT05398705) undertaken by Xinhua Hospital affiliated to Shanghai Jiao Tong University School Of Medicine and Ren Ji Hospital, School of Medicine, Shanghai Jiao Tong University. This is an interventional efficacy and safety, phase II, double-blind, 3-arm study to investigate orally administered high-/low-dose CEP compared with placebo in nonhospitalized asymptomatic or mildly symptomatic adult participants with COVID-19, and the results have not yet been published (US National Library of Medicine, 2022).

While i.v. administration can achieve a fairly high C_max_ value of CEP, it is not a practical treatment option for global distribution and accessibility. However, when administered at a conventional oral dose, CEP C_max_ in humans is too low to be effective against SARS-CoV-2 *in vitro*. Pharmacokinetic studies in animals showed that pulmonary administration significantly improved the absolute bioavailability of CEP, reaching over 64% (as shown in Table 3-D–F). Therefore, delivering CEP through pulmonary administration, such as atomized preparations, nasal sprays, or powders for inhalation, may be a viable option for accelerating its clinical application in the treatment of COVID-19.

As an approved drug that has been used for more than 70 years, CEP is mainly extracted, separated, and purified from plants of the *Menispermaceae* family, including *Stephania japonica (Thunb.) Miers*, *Stephania delavayi Diels*, *Stephania cepharantha Hayata*, *Epigeal stephania root*, and *long Stephania herb*. These plants possess a mature technique for extraction with low prices, and CEP has the advantages of safety, effectiveness, and accessibility. In conclusion, considering its remarkable capacity against SARS-CoV-2 infection *in vitro* and *in vivo*, with the lungs as the fundamental target organ; its good pharmacokinetic profile; and its advantages of safety, effectiveness, and accessibility, CEP is a promising drug candidate for treating COVID-19 (Zhai et al., 2018).
